# Tumor immunotherapy based on tumor-derived heat shock proteins (Review)

**DOI:** 10.3892/ol.2013.1616

**Published:** 2013-10-10

**Authors:** YUNFEI ZHANG, LIANHE ZHENG

**Affiliations:** Department of Orthopedics, Tangdu Hospital, Fourth Military Medical University, Xi’an, Shaanxi 710038, P.R. China

**Keywords:** heat shock proteins, molecular chaperone, immunotherapy, vaccine

## Abstract

Heat shock proteins (HSPs), the most important type of molecular chaperone, are expressed in all eukaryotic cells and have multiple functions, including the folding and unfolding of other proteins and peptides, the transport of proteins and peptides and the support of antigen presentation processes. Due to these important properties, the use of HSPs has been explored as a promising tumor immunotherapy strategy. It has been previously demonstrated that HSP peptide complex (HSP.PC) derived from tumors is the immunogenic entity that elicits powerful antitumor immune responses. Previous animal studies and phase III clinical trials have demonstrated the efficacy, safety and feasibility of HSP-based tumor vaccines. However, the limitations are also apparent and specific alternatives have been developed. The present review focused on the history of HSP-based immunotherapy, the mechanism of its immunogenicity and the previous efforts to promote the efficacy. The current review may be useful for antitumor studies based on the tumor-derived HSPs.

## 1. Introduction

Stress or heat shock proteins (HSPs) were first identified in 1962 as highly-conserved proteins, with expression that is induced by various types of stress (1). Subsequently, it was shown that intracellular HSPs behave as molecular chaperones for other cellular proteins (2,3). HSPs perform a variety of chaperone functions, including the folding and unfolding of nascent polypeptides and proteins (4), the degradation of proteins (5), the transport of proteins and peptides throughout the various cellular compartments (6–8) and the support of antigen presentation processes (9,10). Furthermore, HSPs have been previously shown to have a role in priming immune responses when released from cells in complex with chaperoned antigenic peptides (11). In murine systems, vaccination with purified preparations of selected HSPs (GP96, HSP70 and HSP90) isolated from tumors, but not from normal tissues, leads to protective immunity against the tumors used as the source of the HSPs (12,13). Other than this protection property, the treatment of mice bearing established or residual tumors with such vaccines is effective in reducing the tumor burden and metastasis and prolonging survival (14,15). In clinical trials, autologous tumor-derived HSP peptide complexes (HSP.PCs) have been applied to tumor immunotherapy for the treatment of patients with a variety of advanced malignancies. To date, results from clinical trials, including those from phase III trials, have demonstrated that the immunization strategy induces significant tumor-specific immune responses (16,17). These results have indicated that HSP.PCs have the qualities necessary for a tumor vaccine.

However, the efficacy of the HSP.PC vaccine requires improvement. As a therapeutic tool against established tumors, it is only marginally effective, particularly with a widely metastatic disease (18). In rodent models, autologous tumor-derived HSP.PCs were highly effective in the treatment of a minimal residual disease setting (14,19). However, when animals with a widely metastatic disease were treated, only a small proportion showed any benefit and typically, only a slowing of the tumor growth rate or stabilization of the disease was observed (14,20–22). The success of the clinical trial was also limited, which is consistent with the observations made in animals (16,17). These results indicated that novel HSP-based tumor vaccines, with improved therapeutic potentials, require investigation, and researchers are thus currently making efforts to identify alternatives.

## 2. Location and dual function of HSPs

HSPs are present in all cells in all forms of life and have a dual function depending on their intracellular or extracellular location. Intracellular locations include the cytosol of prokaryotes and the cytosol, nuclei, endoplasmic reticulum (ER), mitochondria and chloroplasts of eukaryotes (2). HSPs normally constitute 5% of the total intracellular proteins. However, under various stresses, such as high temperatures, toxins and oxidative conditions, their levels may rise to >15% (23). Intracellular HSPs have a protective function and allow the cells to exert themselves against environmental stress to survive lethal conditions. Various mechanisms attribute to this cytoprotective function.

In addition to their intracellular location, HSPs have been located on the plasma membrane of malignantly transformed cells, in the extracellular space and on virally/bacterially infected cells. The extracellularly located or membrane-bound HSPs mediate immunological functions through the chaperoning of antigenic peptides. HSPs elicit immune responses modulated by the adaptive or innate immune system, and these immunogenic properties make HSPs good targets for tumor therapy (24).

## 3. Immune responses by HSPs

HSPs originally came to the attention of immunologists primarily as besides their chaperone activity, three additional features characterize HSPs: i) HSPs are efficiently internalized into antigen-presenting cells (APC) by receptor-mediated endocytosis (25); ii) once internalized, HSPs traffic into various cellular compartments where chaperoned peptides are released, processed and made available for assembly to new major histocompatibility complex (MHC) molecules (26); and iii) the internalization of chaperone proteins induces the immune responses, which eventually activates the adaptive (CD8^+^ and CD4^+^ lymphocytes) and innate [natural killer (NK) cell activation and cytokine secretion and maturation of dendritic cells (DCs)] immune responses (27). Due to these properties, HSPs have been described as the ‘Swiss army knife’ fo the immune system (27), and this maxim captures the versatility of HSPs. These features allow HSPs to be exploited to engineer new tumor vaccines and potentially, to generate a full-fledged immune response overcoming tumor escape and interfering with growth and metastasis (28).

## 4. HSP categories

HSPs are classified into >10 families, including small HSPs, HSP40, HSP47, calreticulin, HSP60, HSP70, HSP90 and HSP100, which have various locations and functions (23). Each family is composed of members expressed constitutively or regulated inductively and are targeted to various subcellular compartments. Each HSP family consists of between one to five closely related proteins, although there is little evident amino acid homology between HSP families.

## 5. History of HSP immunity

### Origin of tumor immunotherapy in the early 20th century

The earliest tumor immunotherapy has been traced back to the early 20th century, when physicians attempted to treat tumor patients by vaccination with attenuated tumor cells or their crude extracts. This pioneering study was the origin of the current understanding of the versatile properties of HSPs, which was unclear prior to the 1980s (29). There has been little basis to determine whether these pioneering studies had merit, since the controls commonly used today were absent at that time (30).

### Origin of tumor vaccines in the 1940s–1960s

The vaccines against smallpox and polio have been successfully used, and previous tumor studies have attempted to design tumor vaccines that are immune against the challenge of tumor cells, in the same manner as they are against viruses. With the utilization of inbred strains of mice in the 1940s, interpretable studies of tumor immunity were initiated and the fundamental principles gradually became clear. It was demonstrated that the vaccination of mice with attenuated tumor cells provided protection from a subsequent challenge with live tumor cells, just as a vaccination with attenuated virus immunizes against a subsequent infection with that same virus (31–34). However, in contrast to viruses, tumors may be used to vaccinate against themselves, but not against other tumors. This observation elicited the subsequent studies that led to the emergence of HSPs.

### Purification of HSPs for tumor vaccines in the 1980s

The aforementioned research identified that animals may be specifically vaccinated against their own tumors, but not other tumors, thus promoting the search for the molecules within tumor cells that may be responsible for conferring immunity. This identification predicted that tumors are individually and antigenically distinct and express tumor-specific antigens. Tumor cell lysates were fractionated biochemically and individual fractions were tested for their ability to vaccinate mice against the subsequent challenge of live tumor cells of the same type used for fractionation. Finally, it was demonstrated that fractions that reproducibly protected mice from the tumor challenge contained HSPs (12,29,35). HSPs were initially purified from tumor cells and shown to provide protective immunity in a rat liver cancer model (29). This study was corroborated by two additional studies in 1986 in a mouse fibrosarcoma model (12,35). Consequently, researchers hypothesized that tumor-specific antigens may be heat shock-related proteins. HSP-based tumor vaccines were rapidly and widely tested in animal models. The results were unexpected and in almost every model tested the HSPs demonstrated efficacy; there has been no precedent in tumor immunology (30). This identification inspired researchers to extensively test HSP immunotherapy in cancer patients.

### HSP.PC is the immunogenic entity

As HSP preparations elicit immunity only against the tumors from which they were isolated, instead of antigenically distinct tumors (14), this established an immunological conundrum, since HSPs are among the most highly conserved proteins in evolution. Among human beings and even between humans and mice, HSPs are >95% conserved (2). Therefore, how such conserved molecules, which exist everywhere, were able to elicit specific tumor immunity remained unknown. To answer this conundrum, HSP preparations were isolated from normal tissues and used to immunize the animals, however, no resistance to any tumors was generated (36,37). Therefore, it was hypothesized that HSPs in tumors may harbor mutations that differ among various tumors. However, this hypothesis was excluded by the subsequent investigations.

To test the hypothesis, HSP genes among normal tissues and a variety of tumors were sequenced. Notably, no differences in nucleotide sequences were detected among any of the tissues or tumors (38,39). Therefore, this indicated that it is not HSPs that elicit the tumor antigen-specific immune responses. Advanced investigations were therefore required.

The breakthrough point for solving how HSPs elicit specific tumor immunity depended on the normal function of HSPs inside cells. It is well known that HSPs aid newly synthesized polypeptides in folding into their functional conformation and also aid in the transport of proteins and peptides throughout the various subcellular compartments. Furthermore, it had already been made clear that tumor immunity is mainly mediated by T cells, which recognize MHC molecule-peptide complexes. Therefore, it was indicated that HSP preparations from tumor cells may form complexes with antigenic peptides (39), which activate T cell-mediated responses. Several results supported this indication. Firstly, from the HSP structural aspect, the peptide-binding domain of bacterial HSP70 was crystallized with intact peptide in a readily discernible peptide-binding pocket (40). HSP70 family members possess a domain in the C-terminus that chaperones unfolded proteins and peptides and an N-terminal ATPase domain that controls the opening and closing of the peptide-binding domain. These properties promote the formation of stable complexes with tumor antigen peptides (41). Secondly, a large collection of peptides may be eluted from a homogeneous gp96 preparation, as it is treated with trifluoroacetic acid (9). Thirdly, much stronger support for this indication came when the treatment of a tumor-derived HSP70 preparation with ATP had the following two consequences: i) elution of a wide array of peptide peaks from the HSP70 polypeptide, leaving the polypeptide intact; and ii) rendering of the HSP70 preparation as non-immunogenic and ineffective in immunizing against tumor cells, although the amount of HSP70 polypeptide treated by ATP was equivalent in the untreated HSP70 preparations (42). This was the first demonstration that HSP70, isolated from tumors, was associated with peptides and that the dissociation of peptides from HSP70 resulted in abrogation of the immunogenicity. It was further demonstrated that such peptide-free HSP preparations or free, unchaperoned peptides were not immunogenic (42). Three mammalian HSPs (gp96, HSP90 and HSP70) were purified from tumor cells or pathogen-infected cells, and non-covalently associated peptides were eluted off the HSP with acid or ATP.

Thus, it was confirmed that HSP.PCs are the immunogenic entities isolated from tumorous or infected cells. HSPs chaperone the peptide fingerprint, which includes the antigenic peptides of the cells from which they were isolated. HSP preparations, which harbor the unique repertoire of antigenic peptides that exist in individual tumors, elicit the tumor specific immune responses.

### Summary of previous clinical trials

The earliest clinical trial for HSPs began in 1995 with a phase I pilot study in patients with advanced malignancies that had failed to be treated with previous therapies. This initial autologous HSP vaccine was HSP-peptide complex 96 (HSPPC-96; Oncophage; Antigenics Inc., Lexington, MA, USA), produced from surgically resected tumor tissue and formulated for intradermal or subcutaneous injection (43). The purpose was to determine the proper dosage, to address potential toxicities and side-effects and also to make comparisons with mice in inducing immune responses. The results demonstrated that the autologous tumor-derived HSP vaccine elicited powerful T cell responses against the tumor and did not produce any toxicity, which was consistent with the results obtained in mice. In addition, these results demonstrated the feasibility of the preparation of individual autologous HSP vaccines from each patients’ own tumor. However, since these studies were non-randomized and were only compared with historical controls, the results from these trials were only indicative. Other phase I and II trials in pancreatic or colon cancers have since been completed with similar outcomes. Trials in B lymphoma, chronic myelogenous leukemia, lung cancer and glioma are ongoing (44,45).

In 2001, a study by Castelli *et al* demonstrated in a human system that HSP.PCs purified from tumor cells activate cytotoxic T lymphocyte (CTL) clones specific for defined tumor antigens (7). This study further highlighted the reliable theoretical basis for the use of HSP vaccine in patients.

To further investigate the clinical effect of the autologous HSP vaccine, two phase III trials followed. The first study focused on stage IV melanoma patients and ~322 patients were involved in a randomized, open-label, multicenter phase III trial (16). The patients in the treatment group received HSP vaccine derived from autologous cancers, and the regimen was administration once weekly for the first 4 weeks and subsequently every other week, for as long as the vaccine lasted. The patients in the control group received the physician’s choice of treatment, which consisted of a specific combination of dacarbazine, temozolomide, interleukin (IL)-2 and surgery. The general analysis from the survival plots showed no significant difference between the HSP vaccine treatment and control groups. However, a specific subset analysis was more encouraging, informative and significant. Two critical observations were formed as follows: i) when the vaccine dose increased, patients treated with vaccine received a greater benefit; and ii) with an increasing number of immunizations, the hazard ratios shifted to the left (in favor of vaccine) in M1a and M1b substages, but not M1c substages. However, the success rate for the production of vitespen (four injections are the minimal dosage for vitespan administration) was only 49%, the main reason being the limitated quantity of resected tumor available for HSP isolation.

The vaccine was effective in the early stage of the disease instead of the late stage of the disease. When the HSP regimen was limited to <10 doses, patients with M1a and M1b stages exhibited improved survival rates compared with the control group, which was a statistically significant result. However, no difference was identified between the HSP treatment and control groups for the patients in the M1c substage.

A second phase III trial, in which 728 patients were involved, focused on renal cell carcinoma. To date, this is the largest randomized study for renal cell carcinoma in the adjuvant setting (17). This trial was also a randomized, international, multicenter, open-label study. The HSP vaccine was prepared from surgically removed diseased kidneys. The patients were at high risk for recurrence following nephrectomy, therefore, the endpoint was recurrence-free survival. The patients were randomly distributed in a 1:1 ratio into two groups, the treatment group, nephrectomy plus HSP vaccine; and the control group, nephrectomy alone. The results of this phase III trial were similar to the previously described phase III trial. No difference was identified in recurrence-free survival between patients who received vitespen and patients who did not receive treatment. Specific evidence was identified of an improved recurrence-free survival with vitespen in patients with an earlier stage of the disease (AJCC stages 1 and 2), although the difference between the groups was not statistically significant (P=0.056). Non-protocol-specified post-hoc analyses confirmed that the population of patients identified to correlate with the intermediate-risk category (with stage I/II, high-grade or grade III and T1/2/3a low-grade disease) had significantly fewer recurrence events in the vitespen group than in the observation group (P=0.026). Among the patients at high risk (stage III, T1/2/3a high-grade, T3b, T3c and stage IV) the differences were statistically indistinguishable between the vitespen and observation groups.

From these two randomized phase III clinical trials, several conclusions may be drawn: i) The HSP vaccine was well tolerated and any adverse events were generally mild and expected; ii) the clinical efficacy is associated with the vaccination dose (increased dose and time of vaccination led to increased efficiency) and the disease stage (patients with early stage exhibited apparent benefits in the vaccine treatment group compared with the control group); and iii) later-stage tumors adopt a number of mechanisms to subvert the immune response and become resistant to immunotherapy, offering a potential explanation as to why vaccine therapy appears to have improved function in earlier-stage tumors.

## 6. Mechanisms of immunogenicity of HSP-based vaccines

**Figure 1:**
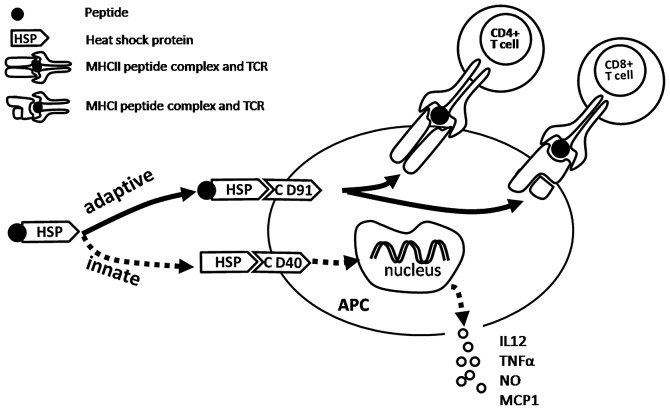
HSP-APC interaction activates adaptive and innate immune responses. The interaction between the HSP-peptide complex and CD91 receptor on the APC leads to stimulation of peptide-specific CD8^+^ and CD4^+^ T lymphocyte responses. The interaction of HSP (no peptide complex) with CD40, CD36, etc., on APCs leads to the non-antigen-specific innate immune responses, including cytokine and chemokine release and DC maturation. HSP, heat shock proteins; APC, antigen-presenting cell; MHC, major histocompatibility complex; IL-12, interleukin 12; MCP-1, monocyte chemoattractant protein-1; NO, nitric oxide; TNFα, tumor necrosis factor α; DC, dendritic cell.

The mechanisms by which HSP.PC immunization elicits potent antitumor effects are becoming clearer. The interaction of HSP.PC with APCs leads, on the one hand, to the presentation of antigenic peptides to CD8^+^ and CD4^+^ T lymphocytes (adaptive immunity) and on the other hand, to a cascade of non-antigen-specific events (innate immunity) that promote immune responses (Fig. 1) (23).

### Activation of adaptive immunity

The fact that immunization with femtomole quantities of antigenic peptides chaperoned by HSPs is effective in eliciting such potent T cell responses is noteworthy (46). By way of comparison, tens to hundreds of micrograms (or tens of nanomoles) of peptides (in a conventional microbial adjuvant) are typically used to immunize and elicit a similar response (47,48). Further investigation was required to analyze how immunization with femtomoles of peptides is effective. There is a general biological principle that extraordinary efficiencies are always achieved through specific receptors. Therefore, researchers hypothesized that HSPs interact with APCs through specific receptors and that such interactions result in the endocytosis of HSP.PC, followed by the processing of peptides and their presentation by MHC molecules (49). The subsequent investigations demonstrated this hypothesis and the answer to this conundrum was identified as CD91. It has been previously confirmed that HSP90, HSP70, calreticulin and gp96 interact with macrophages and DCs through a common receptor, CD91 (50,51).

Once HSP.PC is taken up through CD91, it may enter one or more of several trafficking and processing pathways, which are likely to lead to the stimulation of various T cell responses.

The interaction of HSP.PC with CD91 leads to the internalization of complexes into a non-acidic endosomal compartment and then the complex or the peptide alone is transferred to the cytosol (51–53) by an unknown mechanism. Next, the peptides are processed by the proteasomes and are transported into the ER by the transporters associated with antigen processing (TAP) (51). The peptides are then loaded onto MHC class I molecules. The occupied MHC molecules then pass through the secretory pathway to the cell surface where they interact with the receptors of CD8 T cells.

However, following internalization through the CD91 receptor, a small proportion of HSP.PCs enter an acidic compartment, where the peptide is loaded onto MHC class II molecules, leading to the stimulation of CD4^+^ T cells (54).

Thus, HSP.PC internalized by APCs through CD91 receptors are presented by MHC class I and II molecules, which stimulate the CD8 and CD4 T cells, respectively. CD91 is the main receptor involved in this HSP-based immune response, however, there may be other specific receptors involved, which are currently being investigated (51).

### Activation of innate immunity

It is generally accepted that HSPs, functioning as chaperones for tumor antigens, elicit tumor-specific adaptive immune responses. HSPs also appear to induce innate immune responses in an antigen-independent fashion. Innate responses generated by HSPs may contribute to antitumor immunity. These responses include the cytokine and chemokine release by APCs and T cells and the maturation of DCs. The innate immune responses by the HSP-APC interaction may be summarized as follows (23): i) secretion of inflammatory cytokines, tumor necrosis factor α (TNFα), IL-1β, IL-12 and granulocyte-macrophage colony-stimulating factor (GM-CSF) by macrophages (55) and NK cells, which are stimulated by IL-12, were shown to be required for therapeutic antitumor activity mediated by HSPs (14); ii) secretion of chemokines, including monocyte chemoattractant protein-1 (MCP-1), macrophage inflammatory protein-2 (MIP-2) and regulated upon activation, normal T cells expressed and secreted (RANTES) by T cells (56,57); iii) induction of nitric oxide (NO) production by macrophages and DCs and synthesis of inducible NO (58). The production of NO by HSP-activated APC is likely to have a consequence for the innate control of tumors and infectious diseases. NO released by HSP-activated APC may also provide a layer of immunomodulation of Th cells by necrosis-released HSP. Whereas lower levels of NO are cytoprotective, higher levels are cytotoxic to T cells, particularly Th1 cells (58); iv) maturation of DCs with increased expression of MHC II, B7-2 and CD40 molecules (59); v) migration of DCs from the site of injection of HSPs to the draining lymph nodes. Binder *et al*(60) previously confirmed that the immunization of mice with the HSP, gp96, but not the control proteins, leads to a 5–7 fold enlargement of the draining lymph nodes. This observation uncovered a novel aspect of the HSP-APC interaction and adds to the mechanistic explanation for the unusually high immunogenicity of HSP.PC; and vi) translocation of nuclear factor-κB (NF-κB) into the nuclei of macrophages and DCs, which is a key transcription factor involved in the expression of genes encoding a number of the aforementioned molecules (55).

The previously described immune responses associated with innate immune responses induced by HSPs have independent peptides and play a role in antitumor responses. Previous studies by Udono and Srivastava and Ciupitu *et al* confirmed that prophylactic vaccination with HSPs isolated from normal tissues cause complete tumor rejection in a small proportion of animals, in this case, tumor-specific T cells were not expected to be primed (37,61). Baker-LePain *et al* demonstrated that the rate of tumor growth was slowed without causing complete tumor rejection by HSPs secreted from various irradiated tumor cells and fibroblast lines, in an antigen-non-specific manner (62). In therapeutic settings, tumor-unrelated HSP preparations have been observed to reduce the metastatic burden and prolong the survival rate of mice, albeit in a significantly smaller percentage of mice compared with those treated with tumor-derived HSP preparations (14).

Collectively, while the innate immune response elicited by HSPs contributes to tumor immunity, the adaptive immune responses elicited by HSP-chaperoned tumor-specific peptides are more important in the antitumor immunity.

## 7. Limitations of HSP-based vaccines and alternatives of improvement

Currently, there are >150 medical institutions undertaking basic and clinical research on HSPs. The two largest randomized, open-label, multicenter phase III clinical trials reported in 2008 further confirmed that HSP-based vaccines are safe, effective and clinically feasible, which inspires further research. However, these phase III clinical trials also indicated the limitations of HSP-based vaccines. Firstly, the immunogenicity is not strong enough, since the efficacy was usually observed in early-stage disease or with high-dose vaccination, which is consistent with the results obtained in mice. Secondly, clinical use of HSP vaccines is limited by the yield of tumor tissue from the patients and ~50% of patients do not have adequate tumor cells for the isolation of enough vaccine (16). Therefore, the enhancement of the clinical effect is urgently required.

Certain alternatives have been tested, including HSP-pulsed DCs (18), tumor-derived chaperone-rich cell lysate (CRCL) (63) or a combination with GM-CSF (21,64), which showed improved immunogenicity. To enhance the immunogenicity, specific new methods to prepare the vaccine have been developed, and improved antitumor immune responses have been observed (65,66). A more powerful HSP-based tumor vaccine has been developed from DC/tumor fusion cells, which greatly enhances the immunogenicity of HSP-based vaccines compared with that derived from the tumor. This new vaccine may present an improved treatment against malignant cancer (67,68).

## Conclusions

Tumor-derived HSP-based vaccines have shown great prospect in tumor immune therapy. Various animal studies and clinical trials have demonstrated the efficacy, safety and feasibility of this vaccine. It is now clear that the immunogenicity entity was HSP-peptide complexes derived from tumors and the mechanism was due to activation of adaptive and innate immunity following the interaction of HSP.PC with APCs through receptor mediated endocytosis. However, the limitations are also apparent: i) the immunogenicity is not strong enough and ii) the yield of tumor vaccine was limited. Certain alternatives to improve the vaccine have been made and enhanced responses have been observed. In the future, focus should be on how to improve the immunogenicity and how to increase the bioavailable efficiency of this vaccine. Tumor-derived HSP.PC-based vaccines are a promising vaccination strategy and are likely to provide great help to tumor patients.

## Abbreviations

HSPs: heat shock proteins
HSP.PC: heat shock proteins peptide complex
ER: endoplasmic reticulum
APC: antigen-presenting cells
MHC: major histocompatibility complex
NK: natural killer
DC: dendritic cell
CTL: cytotoxic T lymphocyte
TAP: transporters associated with antigen processing
TNF-α: tumor necrosis factor α
IL-1β: interleukin-1β
GM-CSF: granulocyte-macrophage colony-stimulating factor
MCP-1: monocyte chemoattractant protein-1
MIP-2: macrophage inflammatory protein-2
RANTES: regulated upon activation, normal T cells expressed and secreted
NO: nitric oxide
iNOS: inducible nitric oxide
NF-κB: nuclear factor-κB
CRCL: chaperone-rich cell lysate

## References

[1] Ritossa P (1962). Problems of prophylactic vaccinations of infants. Riv Ist Sieroter Ital.

[2] Lindquist S, Craig EA (1988). The heat-shock proteins. Annu Rev Genet.

[3] Georgopoulos C, Welch WJ (1993). Role of the major heat shock proteins as molecular chaperones. Annu Rev Cell Biol.

[4] Gething MJ, Sambrook J (1992). Protein folding in the cell. Nature.

[5] Parsell DA, Lindquist S (1993). The function of heat-shock proteins in stress tolerance: degradation and reactivation of damaged proteins. Annu Rev Genet.

[6] Bukau B, Deuerling E, Pfund C, Craig EA (2000). Getting newly synthesized proteins into shape. Cell.

[7] Castelli C, Ciupitu AM, Rini F (2001). Human heat shock protein 70 peptide complexes specifically activate antimelanoma T cells. Cancer Res.

[8] Craig EA, Weissman JS, Horwich AL (1994). Heat shock proteins and molecular chaperones: mediators of protein conformation and turnover in the cell. Cell.

[9] Li Z, Srivastava PK (1993). Tumor rejection antigen gp96/grp94 is an ATPase: implications for protein folding and antigen presentation. EMBO J.

[10] Sherman M, Multhoff G (2007). Heat shock proteins in cancer. Ann N Y Acad Sci.

[11] Milani V, Noessner E, Ghose S (2002). Heat shock protein 70: role in antigen presentation and immune stimulation. Int J Hyperthermia.

[12] Srivastava PK, DeLeo AB, Old LJ (1986). Tumor rejection antigens of chemically induced sarcomas of inbred mice. Proc Natl Acad Sci USA.

[13] Vanaja DK, Grossmann ME, Celis E, Young CY (2000). Tumor prevention and antitumor immunity with heat shock protein 70 induced by 15-deoxy-delta12,14-prostaglandin J2 in transgenic adenocarcinoma of mouse prostate cells. Cancer Res.

[14] Tamura Y, Peng P, Liu K, Daou M, Srivastava PK (1997). Immunotherapy of tumors with autologous tumor-derived heat shock protein preparations. Science.

[15] Nicchitta CV (1998). Biochemical, cell biological and immunological issues surrounding the endoplasmic reticulum chaperone GRP94/gp96. Curr Opin Immunol.

[16] Testori A, Richards J, Whitman E (2008). Phase III comparison of vitespen, an autologous tumor-derived heat shock protein gp96 peptide complex vaccine, with physician’s choice of treatment for stage IV melanoma: the C-100-21 Study Group. J Clin Oncol.

[17] Wood C, Srivastava P, Bukowski R (2008). An adjuvant autologous therapeutic vaccine (HSPPC-96; vitespen) versus observation alone for patients at high risk of recurrence after nephrectomy for renal cell carcinoma: a multicentre, open-label, randomised phase III trial. Lancet.

[18] Shinagawa N, Yamazaki K, Tamura Y (2008). Immunotherapy with dendritic cells pulsed with tumor-derived gp96 against murine lung cancer is effective through immune response of CD8+ cytotoxic T lymphocytes and natural killer cells. Cancer Immunol Immunother.

[19] Sato K, Torimoto Y, Tamura Y (2001). Immunotherapy using heat-shock protein preparations of leukemia cells after syngeneic bone marrow transplantation in mice. Blood.

[20] Srivastava PK (2000). Immunotherapy of human cancer: lessons from mice. Nat Immunol.

[21] Kojima T, Yamazaki K, Tamura Y (2003). Granulocyte-macrophage colony-stimulating factor gene-transduced tumor cells combined with tumor-derived gp96 inhibit tumor growth in mice. Hum Gene Ther.

[22] Kovalchin JT, Murthy AS, Horattas MC, Guyton DP, Chandawarkar RY (2001). Determinants of efficacy of immunotherapy with tumor-derived heat shock protein gp96. Cancer Immun.

[23] Srivastava P (2002). Roles of heat-shock proteins in innate and adaptive immunity. Nat Rev Immunol.

[24] Schmitt E, Gehrmann M, Brunet M, Multhoff G, Garrido C (2007). Intracellular and extracellular functions of heat shock proteins: repercussions in cancer therapy. J Leukoc Biol.

[25] Srivastava P (2002). Interaction of heat shock proteins with peptides and antigen presenting cells: chaperoning of the innate and adaptive immune responses. Annu Rev Immunol.

[26] Berwin B, Nicchitta CV (2001). To find the road traveled to tumor immunity: the trafficking itineraries of molecular chaperones in antigen-presenting cells. Traffic.

[27] Schild H, Rammensee HG (2000). gp96 - the immune system’s Swiss army knife. Nat Immunol.

[28] Castelli C, Rivoltini L, Rini F (2004). Heat shock proteins: biological functions and clinical application as personalized vaccines for human cancer. Cancer Immunol Immunother.

[29] Srivastava PK, Das MR (1984). The serologically unique cell surface antigen of Zajdela ascitic hepatoma is also its tumor-associated transplantation antigen. Int J Cancer.

[30] Hoos A, Levey DL (2003). Vaccination with heat shock protein-peptide complexes: from basic science to clinical applications. Expert Rev Vaccines.

[31] Gross L (1943). Intradermal immunization of C3H mice against a sarcoma that originated in an animal of the same line. Cancer Res.

[32] Klein G, Sjogren HO, Klein E, Hellstrom KE (1960). Demonstration of resistance against methylcholanthrene-induced sarcomas in the primary autochthonous host. Cancer Res.

[33] Prehn RT, Main JM (1957). Immunity to methylcholanthrene-induced sarcomas. J Natl Cancer Inst.

[34] Old LJBE, Clarke DA, Carswell EA (1962). Antigenic properties of chemically induced tumors. Ann NY Acad Sci.

[35] Ullrich SJ, Robinson EA, Law LW, Willingham M, Appella E (1986). A mouse tumor-specific transplantation antigen is a heat shock-related protein. Proc Natl Acad Sci USA.

[36] Srivastava PK, Menoret A, Basu S, Binder RJ, McQuade KL (1998). Heat shock proteins come of age: primitive functions acquire new roles in an adaptive world. Immunity.

[37] Udono H, Srivastava PK (1994). Comparison of tumor-specific immunogenicities of stress-induced proteins gp96, hsp90 and hsp70. J Immunol.

[38] Srivastava PK, Chen YT, Old LJ (1987). 5′-structural analysis of genes encoding polymorphic antigens of chemically induced tumors. Proc Natl Acad Sci USA.

[39] Srivastava PK, Maki RG (1991). Stress-induced proteins in immune response to cancer. Curr Top Microbiol Immunol.

[40] Zhu X, Zhao X, Burkholder WF (1996). Structural analysis of substrate binding by the molecular chaperone DnaK. Science.

[41] Bukau B, Horwich AL (1998). The Hsp70 and Hsp60 chaperone machines. Cell.

[42] Udono H, Srivastava PK (1993). Heat shock protein 70-associated peptides elicit specific cancer immunity. J Exp Med.

[43] Janetzki S, Palla D, Rosenhauer V, Lochs H, Lewis JJ, Srivastava PK (2000). Immunization of cancer patients with autologous cancer-derived heat shock protein gp96 preparations: a pilot study. Int J Cancer.

[44] Rivoltini L, Castelli C, Carrabba M (2003). Human tumor-derived heat shock protein 96 mediates in vitro activation and in vivo expansion of melanoma- and colon carcinoma-specific T cells. J Immunol.

[45] Li Z, Qiao Y, Liu B (2005). Combination of imatinib mesylate with autologous leukocyte-derived heat shock protein and chronic myelogenous leukemia. Clin Cancer Res.

[46] Blachere NE, Li Z, Chandawarkar RY (1997). Heat shock protein-peptide complexes, reconstituted in vitro, elicit peptide-specific cytotoxic T lymphocyte response and tumor immunity. J Exp Med.

[47] Martin S, Lappin MB, Kohler J (2000). Peptide immunization indicates that CD8+ T cells are the dominant effector cells in trinitrophenyl-specific contact hypersensitivity. J Invest Dermatol.

[48] Abiru N, Maniatis AK, Yu L (2001). Peptide and major histocompatibility complex-specific breaking of humoral tolerance to native insulin with the B9-23 peptide in diabetes-prone and normal mice. Diabetes.

[49] Srivastava PK, Udono H, Blachere NE, Li Z (1994). Heat shock proteins transfer peptides during antigen processing and CTL priming. Immunogenetics.

[50] Binder RJ, Han DK, Srivastava PK (2000). CD91: a receptor for heat shock protein gp96. Nat Immunol.

[51] Basu S, Binder RJ, Ramalingam T, Srivastava PK (2001). CD91 is a common receptor for heat shock proteins gp96, hsp90, hsp70 and calreticulin. Immunity.

[52] Suto R, Srivastava PK (1995). A mechanism for the specific immunogenicity of heat shock protein-chaperoned peptides. Science.

[53] Singh-Jasuja H, Toes RE, Spee P (2000). Cross-presentation of glycoprotein 96-associated antigens on major histocompatibility complex class I molecules requires receptor-mediated endocytosis. J Exp Med.

[54] Matsutake T, Srivastava PK (2000). CD91 is involved in MHC class II presentation of gp96-chaperoned peptides. Cell Stress Chaperones.

[55] Basu S, Binder RJ, Suto R, Anderson KM, Srivastava PK (2000). Necrotic but not apoptotic cell death releases heat shock proteins, which deliver a partial maturation signal to dendritic cells and activate the NF-kappa B pathway. Int Immunol.

[56] Moré SH, Breloer M, von Bonin A (2001). Eukaryotic heat shock proteins as molecular links in innate and adaptive immune responses: Hsp60-mediated activation of cytotoxic T cells. Int Immunol.

[57] Lehner T, Bergmeier LA, Wang Y (2000). Heat shock proteins generate beta-chemokines which function as innate adjuvants enhancing adaptive immunity. Eur J Immunol.

[58] Panjwani NN, Popova L, Srivastava PK (2002). Heat shock proteins gp96 and hsp70 activate the release of nitric oxide by APCs. J Immunol.

[59] Singh-Jasuja H, Scherer HU, Hilf N (2000). The heat shock protein gp96 induces maturation of dendritic cells and down-regulation of its receptor. Eur J Immunol.

[60] Binder RJ, Anderson KM, Basu S, Srivastava PK (2000). Cutting edge: heat shock protein gp96 induces maturation and migration of CD11c+ cells in vivo. J Immunol.

[61] Ciupitu AM, Petersson M, Kono K, Charo J, Kiessling R (2002). Immunization with heat shock protein 70 from methylcholanthrene-induced sarcomas induces tumor protection correlating with in vitro T cell responses. Cancer Immunol Immunother.

[62] Baker-LePain JC, Sarzotti M, Fields TA, Li CY, Nicchitta CV (2002). GRP94 (gp96) and GRP94 N-terminal geldanamycin binding domain elicit tissue nonrestricted tumor suppression. J Exp Med.

[63] Li G, Zeng Y, Chen X (2007). Human ovarian tumour-derived chaperone-rich cell lysate (CRCL) elicits T cell responses in vitro. Clin Exp Immunol.

[64] Pilla L, Patuzzo R, Rivoltini L (2006). A phase II trial of vaccination with autologous, tumor-derived heat-shock protein peptide complexes Gp96, in combination with GM-CSF and interferon-alpha in metastatic melanoma patients. Cancer Immunol Immunother.

[65] Murshid A, Gong J, Calderwood SK (2013). Purification, preparation and use of chaperone-peptide complexes for tumor immunotherapy. Methods Mol Biol.

[66] Gao Y, Chen X, Gao W, Yang Y, Ma H, Ren X (2012). A new purification method for enhancing the immunogenicity of heat shock protein 70-peptide complexes. Oncol Rep.

[67] Enomoto Y, Bharti A, Khaleque AA (2006). Enhanced immunogenicity of heat shock protein 70 peptide complexes from dendritic cell-tumor fusion cells. J Immunol.

[68] Gong J, Zhang Y, Durfee J (2010). A heat shock protein 70-based vaccine with enhanced immunogenicity for clinical use. J Immunol.

